# MicroRNA 223 Targeting ATG16L1 Affects Microglial Autophagy in the Kainic Acid Model of Temporal Lobe Epilepsy

**DOI:** 10.3389/fneur.2021.704550

**Published:** 2021-07-26

**Authors:** Zhuoyi He, Houminji Chen, Yongsheng Zhong, Qihang Yang, Xuemin Wang, Rongqing Chen, Yanwu Guo

**Affiliations:** ^1^Neurosurgery Center, Department of Functional Neurosurgery, The National Key Clinical Specialty, The Engineering Technology Research Center of Education Ministry of China on Diagnosis and Treatment of Cerebrovascular Disease, Guangdong Provincial Key Laboratory on Brain Function Repair and Regeneration, The Neurosurgery Institute of Guangdong Province, Zhujiang Hospital, Southern Medical University, Guangzhou, China; ^2^Institute of Biomedical and Health Engineering, Shenzhen Institutes of Advanced Technology, Chinese Academy of Sciences, Shenzhen, China; ^3^Key Laboratory of Mental Health of the Ministry of Education, Guangdong-Hong Kong-Macao Greater Bay Area Center for Brain Science and Brain-Inspired Intelligence, Guangdong Province Key Laboratory of Psychiatric Disorders, Department of Neurobiology, School of Basic Medical Sciences, Southern Medical University, Guangzhou, China

**Keywords:** miR-223, microRNAs, epilepsy, hippocampus, microglia, autophagy

## Abstract

This study aimed to explore whether microRNA (miR) 223 affects microglial autophagy by targeting autophagy-related 16-like 1 (ATG16L1) in the kainic acid (KA) model of temporal lobe epilepsy (TLE). The miRNA and mRNA expression levels were quantified using quantitative real-time polymerase chain reaction (qRT-PCR), and the protein expression was investigated using western blotting. A dual-luciferase reporter assay was used to test the direct interaction between miR 223 and ATG16L1. *In situ* hybridization was performed to measure the hippocampal expression of miR 223. We used immunofluorescence staining to assess the expression of ATG16L1 and microtubule-associated protein light chain 3 (LC3) in the murine hippocampal microglia. Inhibitor of miR 223 was utilized to investigate the role of miR 223 in TLE, and the epileptic activity was assessed using electroencephalography (EEG). The autophagosomes were observed by transmission electron microscopy. In patients with TLE, the murine KA model of TLE, and the KA-stimulated BV2 cells, miR 223, and sequestosome 1 (SQSTM1/P62) expressions were remarkably increased, whereas ATG16L1 and LC3 levels were significantly decreased. Using a dual-luciferase reporter assay, ATG16L1 was determined as a direct target of miR 223. Treatment with antagomir 223 alleviated epilepsy, prevented abnormalities in EEG recordings and increased the ATG16L1 and LC3 levels in KA-treated mice. Inhibition of miR 223 induced increased autophagy in BV2 cells upon Rapamycin stimulation. These findings show that miR 223 affects microglial autophagy via ATG16L1 in the KA model of TLE. The miR 223/ATG16L1 pathway may offer a new treatment option for TLE.

## Introduction

Epilepsy is a common neurological disease caused by a sudden abnormal discharge of brain neurons (1, 2). Temporal lobe epilepsy (TLE) is one of the foremost types of epilepsy in adults, and about 30% of patients with TLE are refractory to pharmacological treatment (3). Moreover, there are currently no drugs to prevent the onset and development of TLE (4). Therefore, there is an urgent need to develop new treatment strategies to improve the therapeutic efficacy in this patient population.

Microglia are the most important antigen-presenting cells and constitute the macrophages of the central nervous system (5). Recent studies have suggested that microglia play a critical role in TLE (6). Autophagy is a highly preserved eukaryotic cellular process that recycles long-lived proteins and damaged organelles by lysosomal digestion (7). Recent evidence has shown that autophagy is associated with some neurological disorders, such as Parkinson's disease, Alzheimer's disease, and epilepsy (8–10).

Small non-coding RNAs, namely microRNAs (miRNAs), were altered in the hippocampus of patients with TLE and the brain tissues of epileptic animal models (11). Moreover, miRNAs are widely involved in regulating various physiological activities, including glial cell activation (12) and autophagy (13), which are closely associated with epilepsy. Therefore, miRNAs could be involved in critical regulatory mechanisms of epilepsy, representing novel therapeutic targets. Previous researches have shown that miR 223 is expressed explicitly in some neurological diseases, such as Parkinson's disease, multiple sclerosis, and acute ischemic stroke (14–16). However, the role of miR 223 in TLE remains to be determined.

In summary, the abnormal discharge of neurons during the onset of epilepsy is closely related to miRNAs, microglia, and autophagy. However, the relationships and mechanisms connecting miRNAs, microglia, and autophagy in TLE are still largely unknown. A research showed that miR 223 could regulate microglial autophagy and affect inflammation of the central nervous system by targeting ATG16L1 (17). Moreover, ATG16L1 was found to be associated with neurological diseases (18). Therefore, we investigated the correlation between miR 223, ATG16L1, microglia, and autophagy in TLE and their subsequent effects on this disorder.

## Materials and Methods

### Patients With TLE and Controls

The brain samples of cases included in our study were provided by the Department of Neurosurgery, Zhujiang Hospital, Southern Medical University, China. Our study included a total of 10 patients with TLE and five glioma patients without epilepsy. All patients with TLE showed significant clinical manifestations, and their seizures were recorded by an electroencephalogram (EEG). The control group samples consisted of normal tissues obtained from the expanded resection of gliomas in patients without epilepsy. The two groups of brain tissues included in our experiment were both extracted from the temporal lobe brain tissues. The acquisition and use of human brain tissues were approved by the Medical Ethics Committee of Zhujiang Hospital, Southern Medical University, China (Certificate number: 2016-SJWK-005). All research procedures involving human participants were performed following the National and Institutional requirements and the tenets of the Declaration of Helsinki. Before the analysis, written informed consent was obtained from all patients. These samples were directly cryopreserved at −80°C. The patients' clinical characteristics are presented in Tables 1, 2.

**Table 1:** Clinical characteristic of TLE patients.

**Patients no**.	**Age (Y)**	**Course (Y)**	**Gender (M/F)**	**Resection tissue**	**Pathology**
1	25	5	F	TNl	GTCs
2	37	10	F	TNr	GTCs
3	20	3	M	TNl	GTCs
4	28	7	F	TNr	GTCs
5	23	4	F	TNl	GTCs
6	8	2	M	TNr	GTCs
7	34	9	M	TNr	GTCs
8	35	7	M	TNr	GTCs
9	52	23	F	TNr	GTCs
10	40	14	M	TNr	GTCs

*TLE, temporal lobe epilepsy; Y, years; F, female; M, male; TN, temporal neocortex; l, left; r, right; GTCs, generalized tonic-clonic seizures*.

**Table 2:** Clinical charactristic of the control patients.

**Patients no**.	**Age (Y)**	**Gender (M/F)**	**Resection tissue**	**Etiology diagnosis**	**Pathology**
1	74	F	TNl	Glioma	N
2	36	M	TNr	Glioma	N
3	23	M	TNr	Glioma	N
4	52	M	TNr	Glioma	N
5	33	M	TNr	Glioma	N

*Y, years; F, female; M, male; TN, temporal neocortex; l, left; r, right; N, normal*.

### Animals

Eighty-eight male C57BL/6J mice of 6–8 weeks of age were provided by the Experimental Animal Center of Southern Medical University, China. The experimental animals were fed and housed in a specific pathogen-free animal facility with a 12-h light/dark cycle at the Experimental Animal Center of Zhujiang Hospital, Southern Medical University. Our study was approved by the Animal Ethics Committee of the Southern Medical University, China (Certificate number: LAEC-2020-051). All experimental and animal care procedures were performed in strict accordance with the approved guidelines for animal care.

### Animal Experiments

The mice were divided randomly into a normal control group (*n* = 8) and an experimental group (*n* = 50). The experimental mice were subjected to intracerebroventricular injection of kainic acid (KA; 125 μg/kg) into the right lateral ventricle (bregma coordinates were considered the reference point; abscissa, 1.8 mm; ordinate, 2.0 mm; depth, 2.2 mm). The mice developed epilepsy after 5–60 min. According to the Racine classification, to determine the level of seizures, the recurrence of grade IV–V lasting for 1 h was considered a successful model establishment. The normal control mice received 125 μg/kg of saline, as opposed to KA. In other experiments, at 60 min after the KA injection, 30 mice were subjected to another intracerebroventricular injection of miR 223 antagomir (Antagomir 223) or antagomir negative control (Antagomir-NC) (GenePharma, Shanghai, China) at a dose of 10 nmol/kg.

If the model was successfully established, the mice were then subjected to EEG recordings. The experimental group was randomly divided into six subgroups, and the mice were sacrificed at 6 h, 12 h, 1 d, 2 d, 3 d, or 7 d after status epilepticus. Mouse hippocampal tissues were isolated to perform quantitative real-time polymerase chain reaction (qRT-PCR) assays to investigate miR 223 expression or western blotting assays to assess ATG16L1, light chain 3 (LC3), and P62 levels.

### Mouse Tissue Preparation

For qRT-PCR and western blot analyses, the mice were decapitated at the indicated time after treatment, and hippocampal tissues were rapidly removed and stored at −80°C until further use.

For immunofluorescence assays and *in situ* hybridization, the mice were anesthetized at the indicated time after seizure induction with 1% pentobarbital sodium (50 mg/kg ip). Subsequently, they were perfused with 50 ml of normal saline, followed by 50 ml of 4% paraformaldehyde through the ascending aorta. The mice brains were post-fixed overnight and embedded in paraffin. The brain tissue samples were sectioned at 4 μm thickness and mounted on adhesive slides.

### Cell Culture and Treatments

The microglial cell line BV2 was kindly provided by the Central Laboratory of Zhujiang Hospital, Southern Medical University, China. The BV2 cells were grown in Dulbecco's modified Eagle's medium (Gibco, Gaithersburg, MD, USA) and supplemented with 10% fetal bovine serum and 1% penicillin-streptomycin (Gibco). The BV2 cells were cultured in an incubator at 5% CO_2_ and a temperature of 37°C. The cell line was then incubated with a medium containing 50 or 100 μM KA for 24 h. The BV2 cells were treated with rapamycin for 24 h to induce autophagy. miR 223 inhibitor (In-miR 223) and inhibitor-negative control (In-NC) were provided by GenePharma Company (Shanghai, China). The BV2 cells were transfected with GM siRNA-mate reagent.

### Dual-Luciferase Assay

The miR-target interaction between miR-223 and *ATG16L1* was assessed using a dual-luciferase assay. Briefly, 293T cells (RiboBio, Guangdong, China) were seeded into 96-well plates and co-transfected with luciferase plasmids carrying wild-type or mutant *ATG16L1* (*ATG16L1*-WT and *ATG16L1*-MUT, respectively) and miR 223 mimics (RiboBio). After 48-h of incubation, luciferase reagent and stop reagent were added to the transfected cells according to the manufacturer's instructions.

### RNA Isolation and QRT-PCR Analysis

According to the manufacturer's instructions, total RNA was extracted using the AG RNAex Pro RNA extraction reagent (AG Corp., Toronto, Canada). The Evo M-MLV reverse transcription kit (AG) was used to synthesize cDNA by reverse transcription of total RNA.

The Pro Taq HS SYBR Green premixed qPCR kit (AG) was used for single-step qRT-PCR reactions. To activate SYBR Green, the initial cycle of 95°C was performed for 30 s, followed by reactions with 45 cycles of 95°C for 5 s and 60°C for 30 s, annealing at 65°C for 5 s, and extension at 95°C for 5 s. The human or murine U6 primers were used as internal controls for each specific miR gene amplification. The changes in mRNA levels were quantified using the 2^−Δ*Δct*^ method with internal control. The following primers were used: hsa-miR 223 RT primers, 5′-GTCGTATCCAGTGCAGGGTCCGAGGTATTCGCACTGGATACGACT-3′ and hsa-miR 223 primers, 5′-GCGCGTGTCAGTTTGTCAAAT-3′ and 5′-AGTGCAGGGTCCGAGGTATT-3′; human ATG16L1 primers, 5′-CCTGGAGACGGAGTGCCTAGAC-3′ and 5′-CTTAGTGGCTGCTCTGCTGATGG-3′; human LC3 primers, 5′-GCTTCGCCGACCGCTGTAAG-3′ and 5′-AGCCGTCCTCGTCTTTCTCCTG-3′; mouse ATG16L1 primers, 5′-CAGAGCAGCTACTAAGCGACT-3′ and 5′-AAAAGGGGAGATTCGGACAGA-3′; and mouse LC3 primers, 5′-CGTCCTGGACAAGACCAAGT-3′ and 5′-ATGTGGGTGCCTACGTTCTC-3′. In addition, specific stem-loop multiplex primers used for reverse transcription of mmu-miR 223 were provided by RiboBio Company.

### Western Blot Analysis

For cell protein extraction, cells were analyzed 48 h after treatment. After washing with pre-cooled phosphate-buffered saline (PBS), protein extraction was performed with RIPA lysis buffer (CWBIO, Beijing, China) supplemented with protease and phosphatase inhibitors (CWBIO) by incubating the cells on ice for 5 min and collecting them using a cell scraper. For tissue protein extraction, the samples were treated with RIPA buffer containing protease and phosphatase inhibitors and placed in a grinder for homogenization. The extraction process was similar for cells and tissues. The lysates were centrifuged at 12,000 × *g* for 15 min at 4°C. Then, the supernatant was quantified using the BCA protein assay kit (CWBIO). The supernatant was denatured for 10 min in a 5 × sodium dodecyl sulfate-polyacrylamide gel electrophoresis (SDS-PAGE) loading buffer (Beyotime, Jiangsu, China). An equal amount of protein was separated on a 12% SDS-polyacrylamide gel and then transferred to a polyvinylidene difluoride membrane (Merck Millipore, Burlington, MA, USA). Following blocking in 5% non-fat milk for 1–2 h at 25°C, the membranes were incubated overnight at 4°C with the following primary antibodies: anti-ATG16L1 (NB110-60928SS; RRID:AB_925262; Novusbio, Centennial, CO, USA), anti-ATG16L1 (ab188642; RRID:AB_2891316; Abcam, Cambridge, UK), anti-LC3B (NB100-2220; RRID:AB_10003146; Novusbio), anti-SQSTM1/p62 (#5114; RRID:AB_10624872; Cell Signaling Technology, Beverly, MA, USA), and anti-GAPDH (AB0037; RRID:AB_2891315; Abways Technology, Shanghai, China). After washing three times with tris-buffered saline-Tween solution (TBST; 20 mM Tris base, 135 mM NaCl, 0.1% Tween 20; pH 7.5), the membranes were incubated with secondary antibodies, goat anti-rabbit immunoglobulin-horseradish peroxidase (#7074; RRID:AB_2099233; Cell Signaling Technology), for 1 h. After three further washing steps with TBST, the protein bands were detected with chemiluminescence horseradish peroxidase substrate (Millipore). Protein band intensities were quantified using the ImageJ software (National Institutes of Health, Bethesda, MD, USA).

### *In situ* Hybridization of Mouse Brain Tissue

The mmu-miR 223 *in situ* hybridization detection kit was provided by the China Boster Company (Punjab, Pakistan). The experimental procedure was as follows: paraffin-embedded mouse brain tissues were deparaffinized in xylene and rinsed in ethanol and pure water. Then, a 3% hydrogen peroxide solution was added to the paraffin section and allowed to stand at 25°C for 5–10 min to inactivate the endogenous enzymes, followed by three washes with pure water. Next, freshly diluted pepsin (1 ml of 3% citric acid plus two drops of concentrated pepsin) was added to the slices. After 10 min of digestion at 25°C, the samples for *in situ* hybridization were washed three times with PBS and then once with pure water. The slices were treated with 1% paraformaldehyde (containing 1:1,000 diethylpyrocarbonate), fixed at 25°C for approximately 10 min, and washed three times with pure water. Then, 20 μl of a pre-hybridization solution was added to each slice. Afterward, the slices were placed in a wet box and then in an incubator at 38°C for 2–4 h. After removing the excess liquid, 20 μl of hybridization solution was added to each slice. The slices were placed in a wet box and kept in an incubator at 38°C overnight. After the hybridization, the slices were washed twice for 5 min each with 2 × SSC in water at 37°C, then with 0.5 × SSC at 37°C for 15 min, and finally with 0.2 × SSC at 37°C for 15 min. A sealing liquid was added to the slices, which were placed in an incubator at 37°C for approximately 30 min. After removing the excess liquid, biotinylated mouse anti-digoxigenin was added to the slices. The slices were kept in an incubator at 37°C for 60 min and subsequently washed with PBS four times for 5 min each. SABC-POD was added, then the slices were kept in an incubator at 37°C for approximately 20 min and then washed with PBS three times for 5 min each. After the addition of biotinylated peroxidase, the slices were kept in the incubator at 37°C for another 20 min, before washing them with PBS four times for 5 min each. Color development with 3,3′-diaminobenzidine was performed, and then the sections were washed thoroughly and counter-dyed with hematoxylin. After alcohol dehydration and xylene transparency, a coverslip was mounted.

### Evaluation of the *In situ* Hybridization

The levels of miR-223 in the hippocampus of mice brain tissue were analyzed using the optical density (OD) method, in which an OD higher than the threshold of hybridization signal in the microphotographs was measured using ImageJ. The measurement was performed in the CA1 and CA3 regions.

### Immunofluorescence

Mouse brain paraffin sections (4 μm) were stained with ATG16L1 (ab188642; RRID:AB_2891316; Abcam), LC3B (NB100-2220; RRID:AB_10003146; Novusbio), and AIF1/IBA1 (NB100-1028; RRID:AB_521594; Novusbio) primary antibodies and the secondary antibodies conjugated to either Alexa Fluor 488 (ab150129; RRID:AB_2687506; green color; Abcam) or Alexa Fluor 594 (ab150076; RRID:AB_2782993; red color; Abcam).

### EEG Recordings and Behavioral Observations

EEG recordings of the treated mice were obtained after intracerebroventricular injection. Briefly, the mice were anesthetized with 1% pentobarbital sodium and fixed on a stereotaxic device. The right hippocampus coordinates were marked by the stereotaxic device, and three metal electrodes were installed on the mouse skull for EEG recording. The recording electrode was located above the contralateral hippocampus (bregma coordinates were considered the reference point; abscissa, −1.8 mm; ordinate, −2.0 mm). The reference electrode was located on the frontal bone, and the ground electrode was located on the occipital bone. All electrodes were covered with resin, and the wound was disinfected with iodine to prevent infection.

The mice were allowed to recover for 1 week; afterward, they were anesthetized and fixed on the stereotaxic device. An EEG recorder (Solar Corp., Makati, Philippines) was used to acquire the EEG signal. Initially, the EEG was recorded for 10 min as the baseline. Then, KA or saline was injected into the right hippocampus with a microsyringe. The EEG was continuously recorded for 2 h after the microsyringe was withdrawn. The EEG recordings were analyzed using MATLAB, and the number of seizures, their duration, and EEG power spectral density of mice after drug administration were used to prove the therapeutic effect of miR 223 antagomir administration. After that, seizure induction in mice was observed and graded as I–V according to the Racine scale (19).

The EEG data were preprocessed with a band-pass filter (14–100 Hz). Epileptic spikes were counted by optimizing the automated seizure detection algorithm in AcqKnowledge 4.2 (Biopac System, Goleta, CA, USA) during 2 h in mice after KA or saline injection and treated with antagomir 223 or antagomir-NC. Spikes were defined as a sharp deflection with an amplitude more than 2–5 times the background activity and lasting 40–60 ms (20).

### Transmission Electron Microscopy

The cultured cells were fixed with 2.5% glutaraldehyde at 25°C for about 5 min, then collected and centrifuged for 2 min. Then, new 2.5% glutaraldehyde was added to fix the cells after the fixative was discarded. After the cells were fixed at 25°C and protected from light for 30 min, they were transferred to 4°C for storage. The sample was handed over to Servicebio Company for subsequent processing and photographed with an HT7700 transmission electron microscope (Hitachi, Japan).

### Statistical Analysis

Statistical analyses were performed using the IBM SPSS Statistics 20 (IBM Corp., Armonk, NY, USA). All data were expressed as means ± standard deviations and were generated using GraphPad Prism (GraphPad Inc., San Diego, CA, USA). The difference between the two groups was assessed using unpaired Student's *t*-test. A value of *p* < 0.05 was considered to be statistically significant.

## Results

### miR 223 Levels in TLE Patients, the Mouse TLE Model, and KA-Treated BV2 Cells

**Figure 1:**
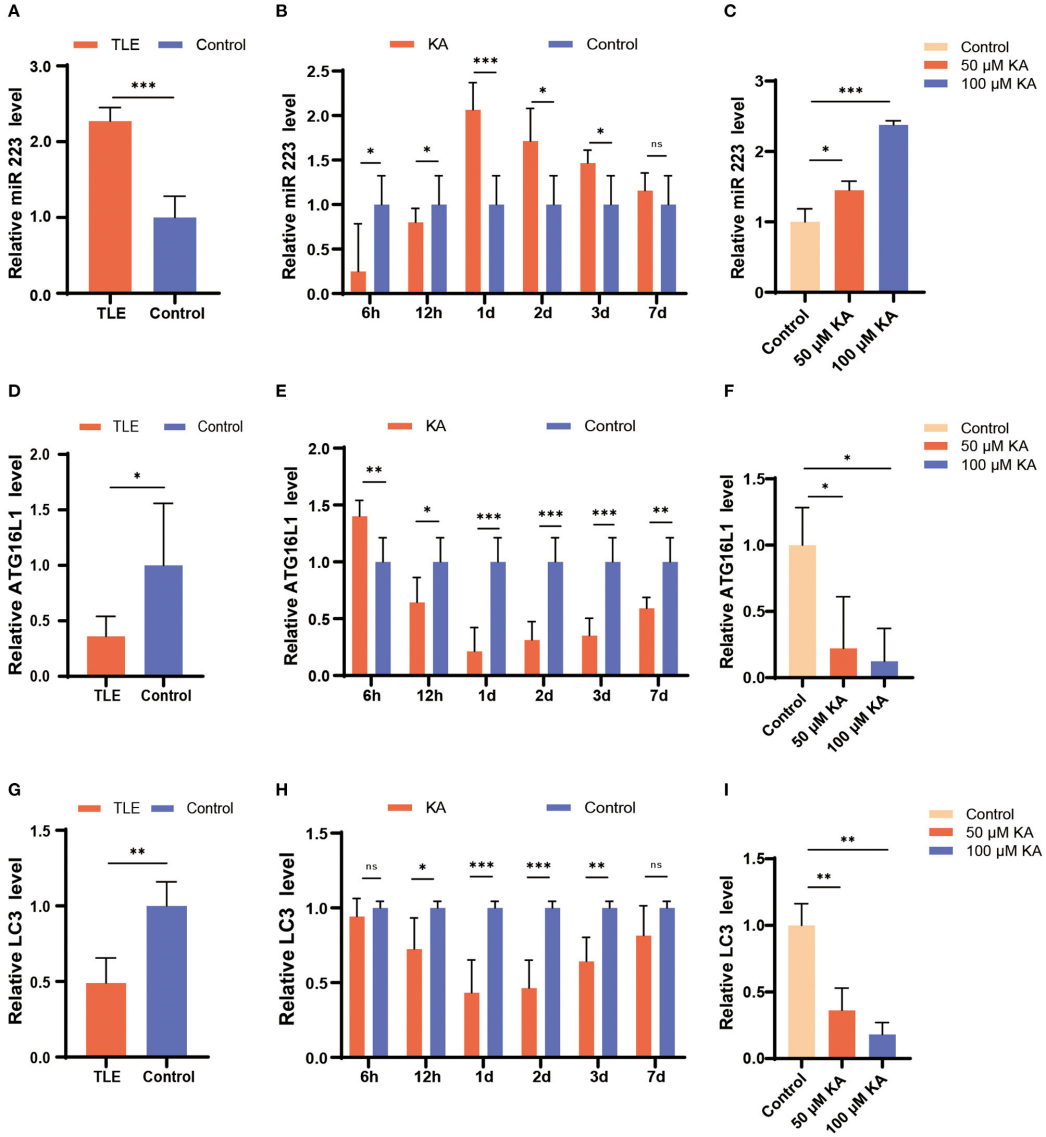
Changes in miR 223, ATG16L1, and LC3 expression patterns in TLE patients, the mouse TLE model, and BV2 cells. **(A)** Specimens from patients with TLE (*n* = 10) and controls (*n* = 5) were analyzed for miR 223 levels. **(B)** C57BL/6J mice were intracerebroventricularly injected with KA or saline and sacrificed at 6 h, 12 h, 1 d, 2 d, 3 d, or 7 d after the treatment. The mice hippocampal tissues were analyzed to assess the miR 223 levels using qRT-PCR (*n* = 6 per group). **(C)** A BV2 cell line was treated with various concentrations of KA for 24 h. The miR 223 levels in the cells were analyzed using qRT-PCR (*n* = 3 per group). **(D–F)** qRT-PCR measurement of *ATG16L1* mRNA levels in samples of TLE patients **(D)**, mouse hippocampal tissue **(E)**, and BV2 cells stimulated with KA **(F)**. **(G–I)** qRT-PCR measurement of *LC3* mRNA levels in samples of TLE patients **(G)**, mouse hippocampal tissue **(H)**, and BV2 cells stimulated with KA **(I)**. Statistically significant differences were determined using the unpaired *t*-test. Data are presented as means ± *SD*; *p < 0.05; **p < 0.01; ***p < 0.001; ns, not significant. miR, microRNA; TLE, temporal lobe epilepsy; qRT-PCR, quantitative real-time polymerase chain reaction; SD, standard deviation; KA, kainic acid; ns, not significant; LC3, light chain 3.

First, we investigated the expression levels of miR 223 in TLE patients using qRT-PCR and found that they were significantly increased in patients with TLE compared to the controls (*p* < 0.001; Figure 1A).

**Figure 2:**
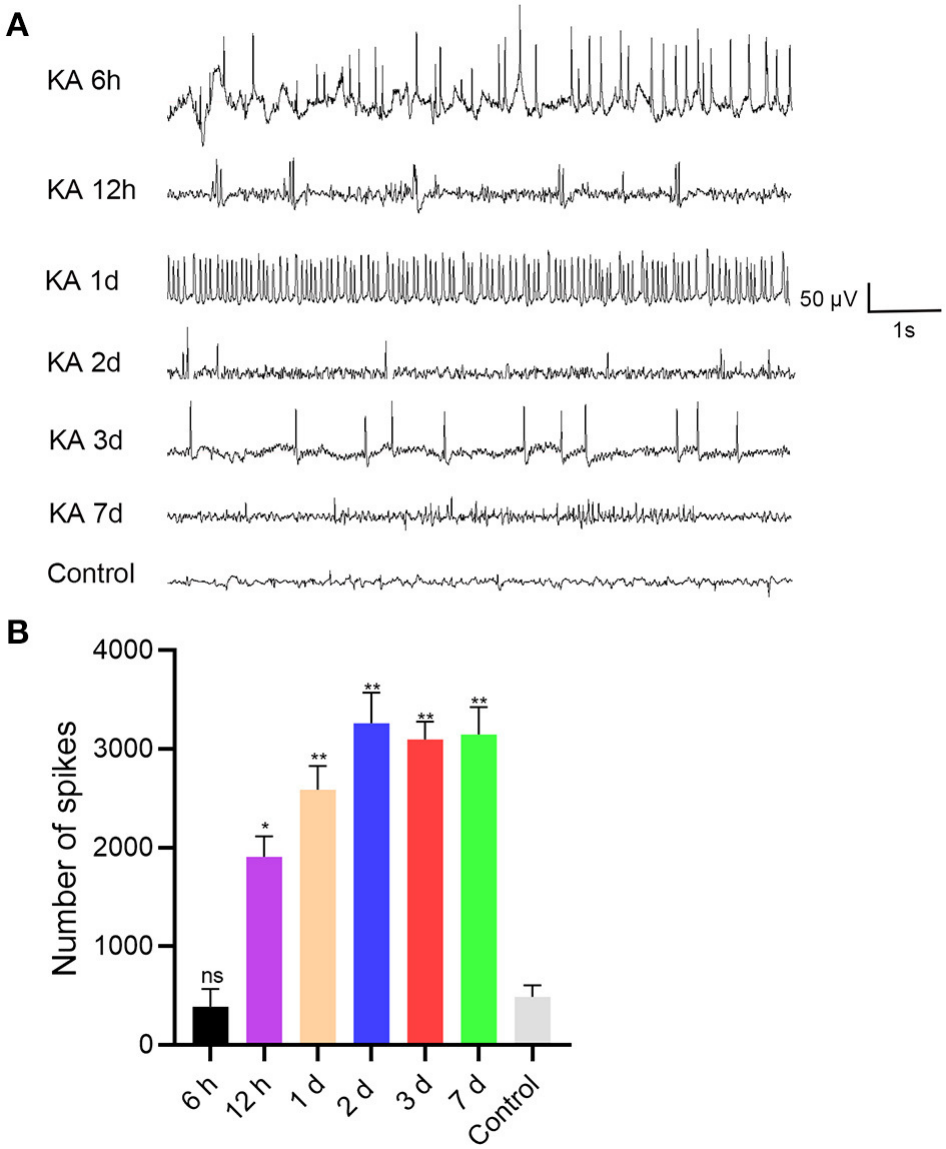
EEG was recorded after intracranial injection of KA or saline in C57BL/6J mice (*n* = 6 per group). **(A)** EEG analysis was performed on the mice, and representative images were displayed. **(B)** The number of seizure spikes in EEG. Statistically significant differences were determined using the unpaired *t*-test. Data are presented as means ± *SD*; *p < 0.05; **p < 0.01; ns, not significant. EEG, electroencephalography; KA, kainic acid; ns, not significant.

To determine the potential role of miR 223 in the mouse model of TLE, we established animal models by unilateral intracerebroventricular injection of KA into the murine brain. The results showed that the KA-treated mice exhibited generalized tonic-clonic seizures and reached grades IV–V of the Racine scale. EEG recordings at 6 h, 12 h, 1 d, 2 d, 3 d, and 7 d after status epilepticus determined high-amplitude and high-frequency discharges in the KA-treated mice compared to the control group (Figure 2A). The number of seizure spikes in all KA groups was significantly increased compared to controls, except in the KA 6 h group (Figure 2B). KA-treated and control mice were sacrificed at the indicated time after treatment. Then, the miR 223 levels in the mice hippocampus were determined using qRT-PCR. The results showed that miR 223 expression was increased in the epileptic hippocampus of mice at all six-time points compared to the control mice. The miR 223 levels were notably higher at 1 d after induction of epilepsy and fell to lower levels after 7 d, although still higher than those in the saline-treated controls (Figure 1B). Moreover, the effect of KA treatment on the increased levels of miR 223 was confirmed in the BV2 cell line (Figure 1C).

In contrast, the expression levels of *ATG16L1* and *LC3* mRNAs followed an opposite trend; they were found to be significantly decreased in TLE patients, the murine epileptogenic hippocampus, and BV2 cells stimulated with KA (Figures 1D–I).

### Expression Patterns of ATG16L1, LC3, and P62 in TLE Patients, the Mouse TLE Model, and KA-Treated BV2 Cells

**Figure 3:**
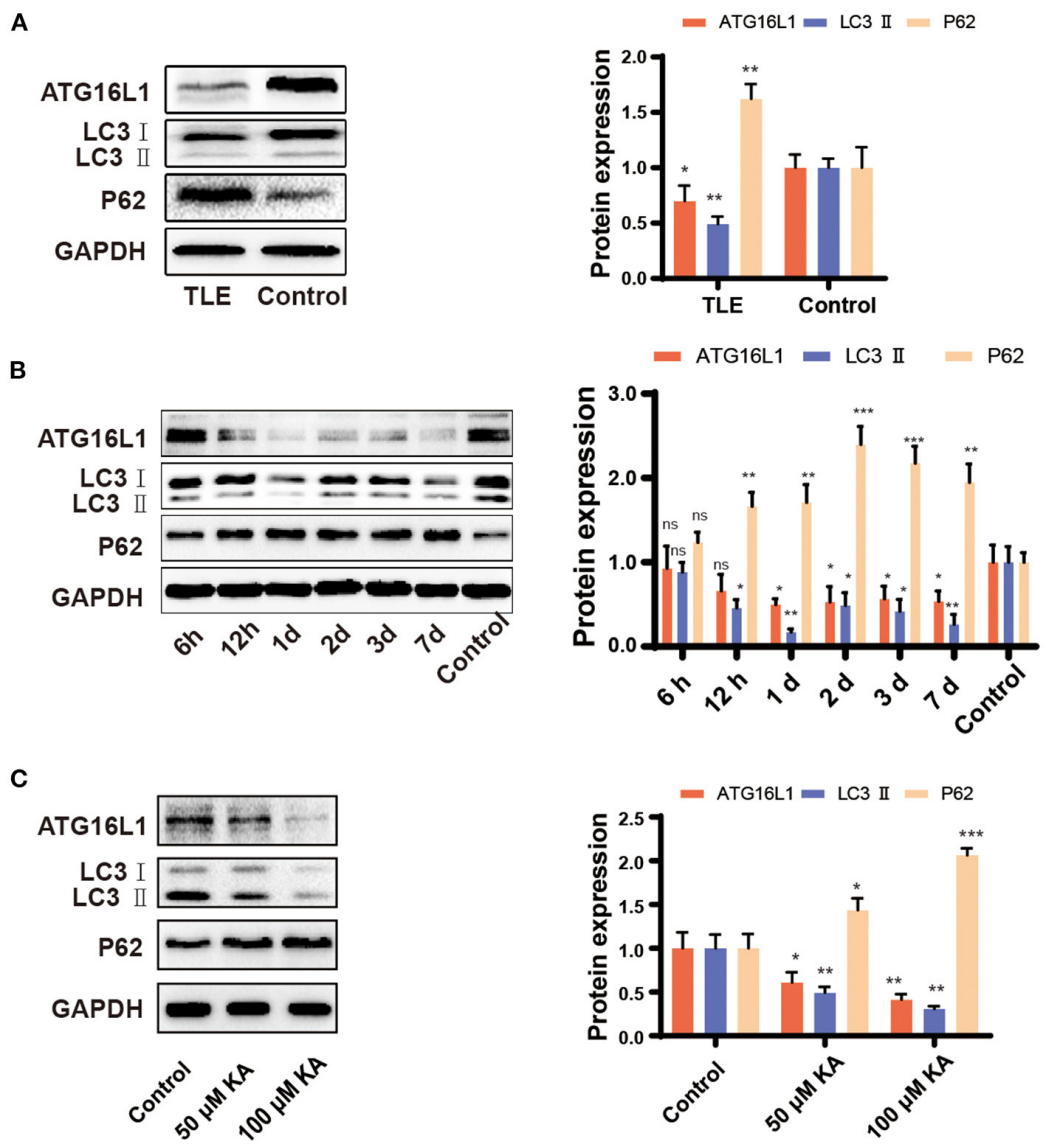
Expression pattern of ATG16L1, LC3, and P62 in TLE patients, the mouse TLE model, and BV2 cells. **(A)** Temporal lobe tissues obtained from patients with TLE and controls, **(B)** mouse hippocampal tissues, and **(C)** BV2 cell lines were analyzed to assess the ATG16L1, LC3, and P62 protein expression levels through western blotting. Statistically significant differences were determined using the unpaired *t*-test. Data are presented as means ± *SD*. *p < 0.05; **p < 0.01; ***p < 0.001; ns, not significant (*n* = 3 per group). miR, microRNA; TLE, temporal lobe epilepsy; *SD*, standard deviation; KA, kainic acid; ns, not significant; LC3, light chain 3.

The qRT-PCR findings for *ATG16L1* and *LC3* were confirmed by western blotting, which revealed changes similar to those observed in the gene expression. Moreover, we found that the expression of the protein P62 was increased in patients with TLE, the epileptic hippocampus of mice, and KA-stimulated BV2 cells (Figures 3A–C).

### Association Between the *ATG16L1* Gene and miR 223

**Figure 4:**
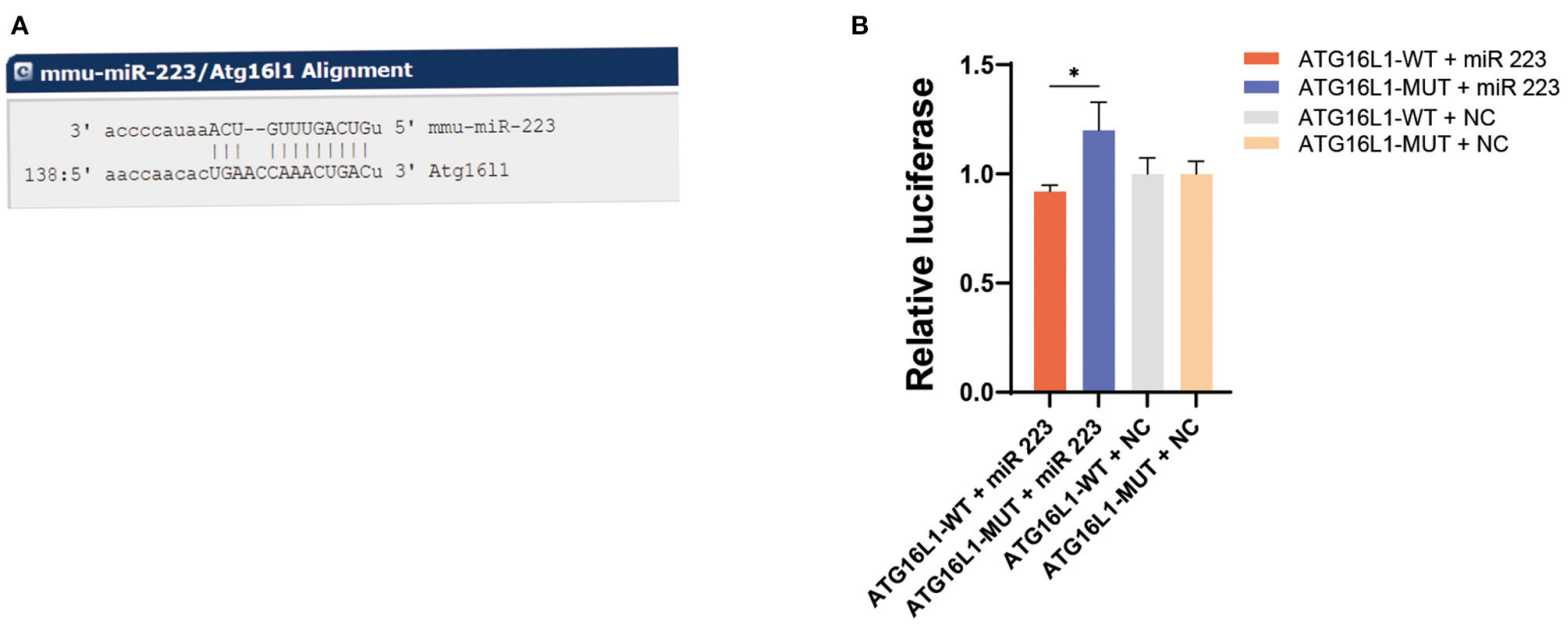
*ATG16L1* gene is a direct target of miR 223. **(A)** The site for miR 223 binding to ATG16L1 was predicted by (http://www.microRNA.org) (http://www.microrna.org/microrna/home.do). **(B)** miR-223 regulates *ATG16L1* expression, as shown by the dual-luciferase reporter assay in 293T cells. Statistically significant differences were determined using the unpaired *t*-test. Data are presented as means ± *SD*; *p < 0.05 (*n* = 3 per group). miR, microRNA; *SD*, standard deviation.

Bioinformatic analysis using the (http://www.microRNA.org) (http://www.microrna.org/microrna/home.do) website determined that *ATG16L1* is a downstream target gene of miR 223 (Figure 4A). To determine precisely the role of miR 223 on *ATG16L1*, we inserted the luciferase reporter and the wild-type or mutant 3′UTR sequence of *ATG16L1* into a vector to construct recombinant plasmids of *ATG16L1*-WT and *ATG16L1*-MUT. A dual-luciferase reporter assay showed that the luciferase activity of miR 223 was significantly increased in the *ATG16L1*-MUT + miR 223 group compared to the *ATG16L1*-WT + miR 223 group (*p* < 0.05; Figure 4B). These results indicate that miR 223 is likely to have significant interaction with the mutated site of the *ATG16L1* 3′UTR sequence.

### *In vivo* Depletion of miR 223 Using an Antagomir

**Figure 5:**
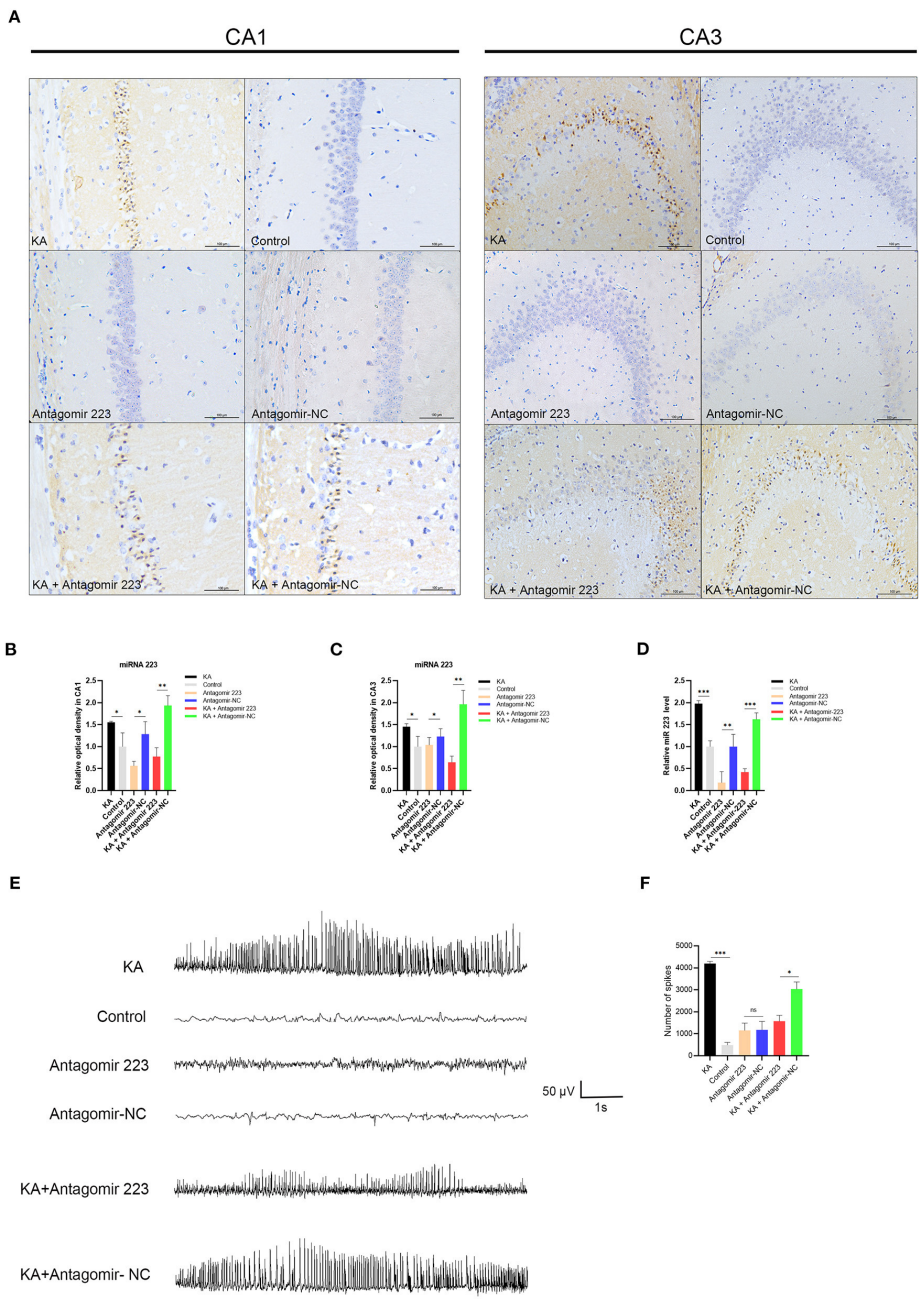
Effects of antagomir 223 on hippocampal miR 223 levels and seizure-like EEG findings. Mice were injected intracerebroventricularly with antagomir 223 or antagomir-NC alone or at 60 min after status epilepticus. **(A)** *In situ* hybridization of miR-223 in the hippocampal tissue. High expression levels of miR-223 were observed in the CA1 and CA3 regions of the KA and KA+Antagomir-NC groups, while the miR 223 hybridization signals in these regions were weaker in the KA+Antagomir-223 group. Scale bar = 100 μm. **(B,C)** OD analysis revealed higher expression levels in the CA1 and CA3 regions of KA-treated mice relative to control. In the KA+Antagomir-223 group, the expression of miR-223 was reduced in the CA1 and CA3 regions (*n* = 3 per group). **(D)** The hippocampal tissues were analyzed to assess the miR-223 levels using qRT-PCR (*n* = 6 per group). **(E)** Representative EEG images of mice, taken 15 min after the final injection. **(F)** The number of seizure spikes in EEG. Statistically significant differences were determined using the unpaired *t*-test. Data are presented as means ± *SD*; *p < 0.05; **p < 0.01; ***p < 0.001; ns, not significant. EEG, electroencephalography; KA, kainic acid; OD, optical density; ns, not significant; SD, standard deviation.

Next, we investigated the function of miR 223 *in vivo* through intracerebroventricular injection of miR 223 antagomir (antagomir 223) or antagomir NC into KA-treated mice to reduce the brain levels of miR 223. We assessed the expression and distribution of miR 223 using *in situ* hybridization. The results indicated that the pattern of miR 223 expression was different 1 d after injection in KA-injected mice compared to the control group, with stronger hybridization signals observed in the CA1 and CA3 regions. However, when we injected miR 223 antagomir into the KA-treated mice, the hybridization signals of miR 223 in the CA1 and CA3 regions were weaker, and the expression level of miR 223 was markedly reduced (Figure 5A). The OD analysis of the CA1 and CA3 regions showed that the expression levels of miR 223 were approximately 1.5-fold higher in KA-treated mice than in control animals. When we injected miR 223 antagomir into the KA-treated mice, the expression of miR 223 in the CA1 and CA3 regions was reduced by half (Figures 5B,C). Also, the obtained qRT-PCR results showed that injection of miR 223 antagomir markedly reduced the hippocampal miR 223 levels upon KA treatment (*p* < 0.001; Figure 5D). The EEG results showed that treatment with miR 223 antagomir markedly reduced seizure severity and decreased the percentage of abnormal EEG recordings compared to the high-amplitude and high-frequency recordings in KA-treated mice without miR 223 antagomir injection (Figure 5E). Compared with the KA + Antagomir NC group, the number of seizure spikes in the KA + Antagomir 223 group was significantly reduced (Figure 5F).

### Effects of miR 223 Silencing on *ATG16L1* Expression and Microglial Autophagy

**Figure 6:**
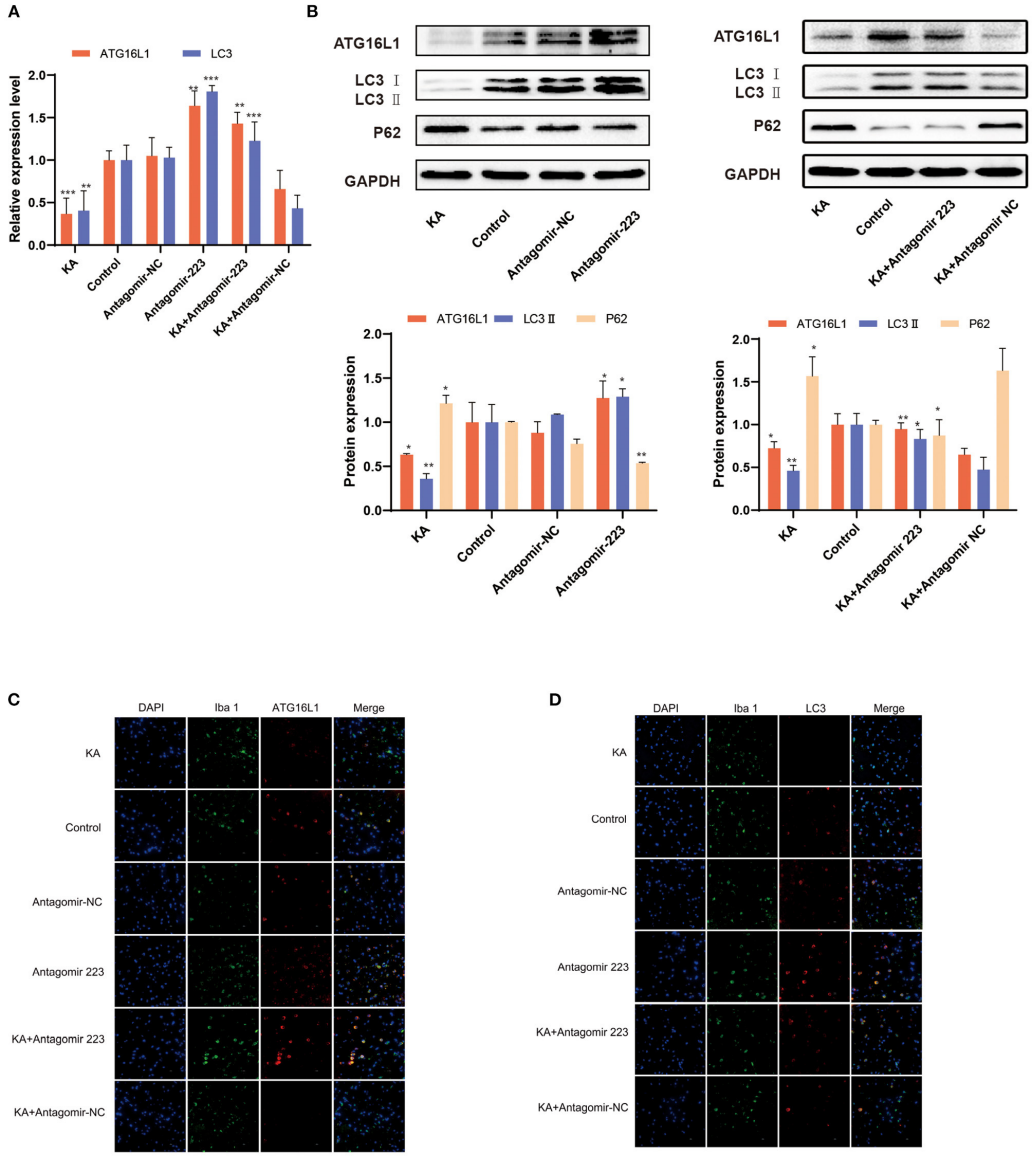
Effects of miR 223 silencing on *ATG16L1* expression and microglial autophagy. **(A)** The hippocampal tissues were analyzed for ATG16L1 and LC3 levels using qRT-PCR (*n* = 6 per group). **(B)** The hippocampal tissues were analyzed to assess the ATG16L1, LC3, and P62 protein expression levels through western blotting. **(C,D)** ATG16L1 levels **(C)** and autophagy **(D)** were measured in the mice hippocampus. The cell-type specificity of ATG16L1 and LC3 (TxRed, red) was established by double-fluorescent immunolabeling with the microglia-specific marker Iba 1 (FITC, green). The nuclei were stained with DAPI (blue). Statistically significant differences were determined using the unpaired *t*-test. Data are presented as means ± *SD*; *p < 0.05; **p < 0.01; ***p < 0.001. Scale bar = 10 μm. miR, microRNA; TLE, temporal lobe epilepsy; SD, standard deviation; LC3, light chain 3.

When the KA-treated mice were injected with miR 223 antagomir, the *ATG16L1* and *LC3* expression levels significantly increased (Figure 6A). We confirmed these results by detecting the expression of ATG16L1, LC3, and P62 proteins by western blot, which showed the same changes for proteins as for the corresponding mRNAs (Figure 6B). There were considerably lower levels of ATG16L1 and LC3 proteins in microglial cells of the KA group than in those of the control group. In contrast, the expression levels of ATG16L1 and LC3 were increased in the microglia of the KA + miR 223 antagomir group (Figures 6C,D).

### Inhibition of miR 223 Expression Promoting Autophagy in BV2 Microglial Cells

**Figure 7:**
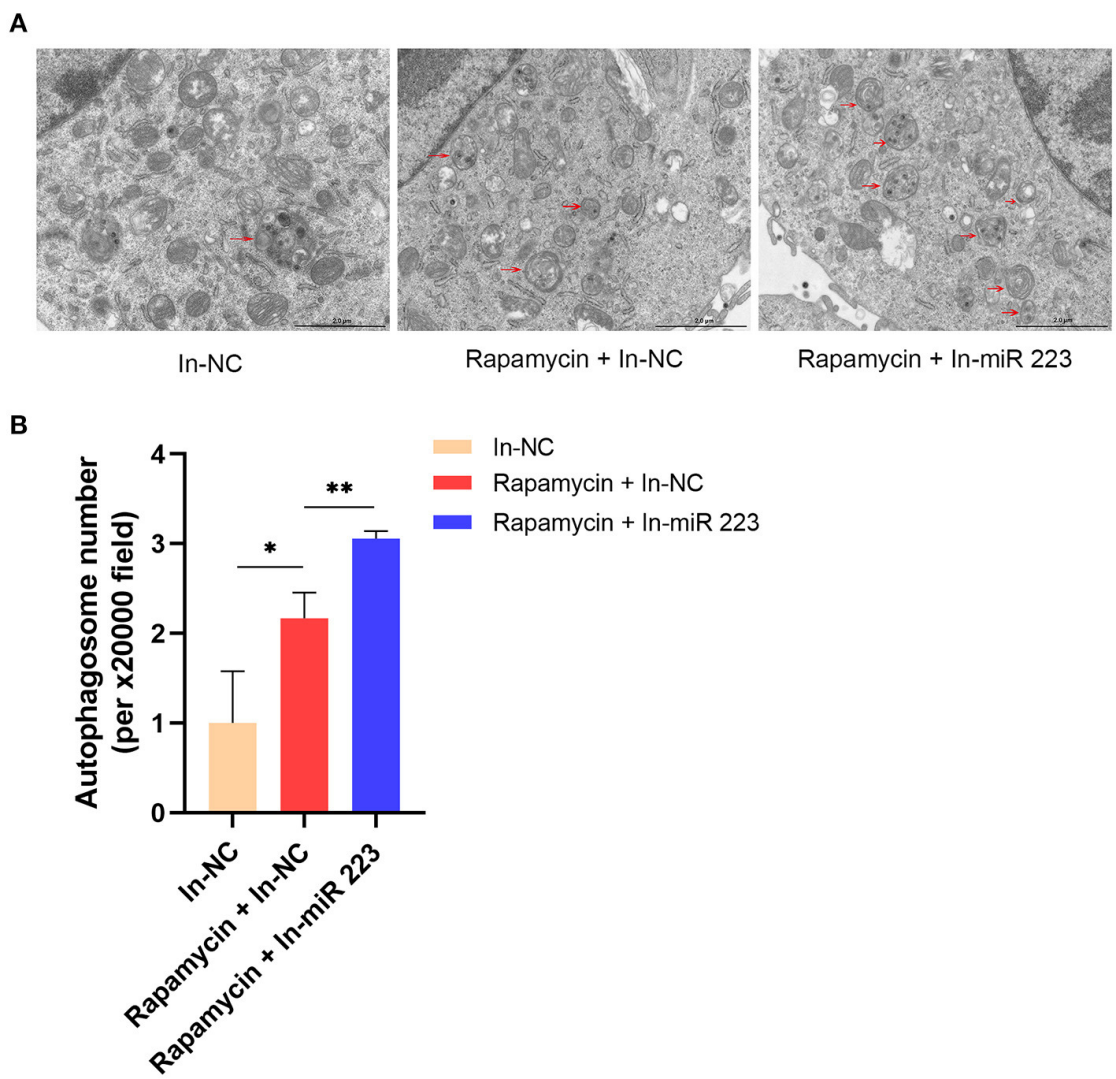
Inhibition of miR 223 increased autophagy in BV2 cells. **(A)** Inhibition of miR 223 induced increased autophagy in BV2 cells upon Rapamycin stimulation. **(B)** Quantitative analysis of autophagosome number. Statistically significant differences were determined using the unpaired *t*-test. Data are presented as means ± *SD*; *p < 0.05; **p < 0.01. Scale bars: 2.0 μm.

We treated BV2 microglial cells with a miR 223 inhibitor *in vitro* to prove that miR 223 can affect microglial autophagy. The results showed that inhibition of miR 223 induced increased autophagy in BV2 cells upon Rapamycin stimulation (Figures 7A,B).

## Discussion

In this study, we explored the expression levels of miR 223 in patients with TLE and a murine KA model of TLE and the regulatory role of miR 223 in BV2 cells after KA stimulation. We found a marked increase in the expression of miR 223 in TLE patients, KA-treated mice, and BV2 cells stimulated with KA. Furthermore, our results demonstrated that the *ATG16L1* gene is a direct target of miR 223, and miR 223 can negatively regulate *ATG16L1* gene expression and affect microglial autophagy.

The analysis of brain tissue samples obtained from patients with TLE suggests that a large-scale dysregulation of the gene expression accompanies TLE initiation and progression. This dysregulation affects the entire network of genes that regulate pathways involving gliosis, inflammation, and neuronal function (21). Future treatment strategies for TLE may target crucial nodes in these pathways. In epilepsy research, miRNAs have gained interest as several types are dysregulated in human TLE (22, 23). miR 223 is an evolutionarily preserved anti-inflammatory miRNA (24), enriched in the microglia (25), though its role in TLE remains to be determined. miR 223 expression is dysregulated in various neuropathologies (26–28), and increased expression was found in the brain of epileptic mice (29). In this study, we found a significant increase in miR 223 levels in patients with TLE. Next, we explored the potential of miR 223 as a treatment target for TLE in mice. During the occurrence of KA-induced epilepsy, the level of miR 223 in the murine hippocampi increased with time, reaching the maximum level on the first day after epilepsy induction, significantly higher than in controls. These results support previous report (30) and microarray finding (29) that miR 223 is up-regulated in human and mice TLE. In agreement with the human and murine results, BV2 cells treated with KA also showed increased levels of miR 223. Importantly, we found that inhibiting the expression of miR 223 with antagomir 223 reduced seizure-like EEG findings and epileptic seizures in mice. Our results showed that miR 223 plays significant roles in the formation and development of TLE. Thus, we hypothesized that miR 223 could be a potential target for TLE treatment.

One study reported that miR 223 deficiency significantly ameliorated inflammation of the central nervous system and increased autophagy in brain microglial cells by targeting *ATG16L1* (17). However, the relationship between miR 223 and *ATG16L1* in TLE and its regulatory mechanisms have not been studied before. We previously discovered the role of miR 223 in patients with TLE and the murine KA model of TLE, so we hypothesized that miR 223 might regulate *ATG16L1* in this epileptic model and, thus, affect microglial autophagy and TLE. In further research, we found that the increased levels of miR 223 observed in patients with TLE and the epileptic hippocampus of mice accompanied the downregulation of *ATG16L1* expression by using qRT-PCR and western blotting. In the microglial cells stimulated with KA, the expression of miR 223 increased significantly, while *ATG16L1* expression decreased. In microglial cells stimulated with high concentrations of KA, the expression levels of miR 223 and ATG16L1 changed more significantly. Moreover, *ATG16L1* was determined to be a direct target of miR 223 based on a dual-luciferase reporter assay results. We also confirmed that inhibition of miR 223 with antagomir 223 could increase the expression of *ATG16L1*. In addition, we found that the knockdown of miR 223 with antagomir 223 *in vivo* alleviated epilepsy and decreased abnormal EEG recordings. Therefore, our results suggest that miR 223 participates in the onset and development of TLE by targeting *ATG16L1*.

Recent evidence suggests that autophagy is related to epileptogenic mechanisms (31). LC3 conjugation is used as the molecular marker for autophagosome production and accumulation. Furthermore, autophagy inhibition results in an increase in the number and size of P62 bodies and P62 protein levels (32). We found that the downregulation of *ATG16L1* in patients with TLE, epileptic mice, and KA-stimulated BV2 cells was accompanied by decreased LC3 levels, whereas there was an increase in P62 expression. Moreover, immunofluorescence results showed that *ATG16L1* and microglial autophagy were reduced in the hippocampus of epileptic mice, whereas epileptic mice that had been injected intracranially with antagomir 223 exhibited the opposite effect. Transmission electron microscopy results also showed that inhibiting the expression of miR 223 can increase microglial autophagy. In summary, these results suggest that miR 223 regulates microglial autophagy by targeting *ATG16L1* during TLE onset and development.

The novelty of our study lies in the first demonstration of the role of miR 223 in TLE and the connection of miRNA, microglia, autophagy, and TLE. This study had some limitations. Our study observed the changing trend of miR 223, ATG16L1, and microglia autophagy levels after acute epileptic seizures. The consequences of these trends and the underlying mechanisms of these changes need to be further studied. TLE is a chronic disease, and we should continue to study the role of these factors in chronic TLE models.

At present, few studies have identified specific miRNAs that can be used as therapeutic targets for diseases. Especially, one study found that silencing miR 134 has neuroprotective effects and inhibits seizures (33). In our study, we found that treatment with antagomir 223 reduced epileptic seizures. Therefore, we hypothesized that, in addition to miR 134 silencing, the silencing of miR 223 might provide another strategy to ameliorate TLE.

## Data Availability Statement

The original contributions presented in the study are included in the article/supplementary material, further inquiries can be directed to the corresponding author/s.

## Ethics Statement

The studies involving human participants were reviewed and approved by the Medical Ethics Committee of Zhujiang Hospital, Southern Medical University, China. The patients/participants provided their written informed consent to participate in this study. The animal study was reviewed and approved by the Animal Ethics Committee of the Southern Medical University, China. Written informed consent was obtained from the individual(s) for the publication of any potentially identifiable images or data included in this article.

## Author Contributions

ZH, XW, RC, and YG designed experiments. ZH, HC, and YZ conducted the experiments. QY analyzed EEG data. ZH and YG wrote and revised the manuscript. All authors contributed to the article and approved the submitted version.

## Conflict of Interest

The authors declare that the research was conducted in the absence of any commercial or financial relationships that could be construed as a potential conflict of interest.

## Publisher's Note

All claims expressed in this article are solely those of the authors and do not necessarily represent those of their affiliated organizations, or those of the publisher, the editors and the reviewers. Any product that may be evaluated in this article, or claim that may be made by its manufacturer, is not guaranteed or endorsed by the publisher.
